# Thalidomide in the Treatment of Sweet's Syndrome and Eosinophilic Folliculitis Associated With Immune Reconstitution Inflammatory Syndrome

**DOI:** 10.3389/fmed.2019.00343

**Published:** 2020-01-21

**Authors:** Rong-Jing Dong, Shi-Zhen Huang, Pratishtha Upadhyay, Samip Shrestha, Ya-Jie Zhai, Yu-Ye Li

**Affiliations:** ^1^Department of Dermatology and Venereology, First Affiliated Hospital of Kunming Medical University, Kunming, China; ^2^Yunnan Provincial Hospital of Infectious Disease/Yunnan AIDS Care Center (YNACC), Anning, China; ^3^Department of Medical Imaging, First Affiliated Hospital of Kunming Medical University, Kunming, China; ^4^Department of Dermatology and Venereology, Jining Second People's Hospital, Jining, China

**Keywords:** AIDS, thalidomide, sweet's syndrome, eosinophilic folliculitis, IRIS

## Abstract

Sweet's syndrome and eosinophilic folliculitis are aseptic inflammatory dermatitis mainly because of infiltrated neutrophils and eosinophils on skin, respectively. These diseases rarely overlap or coexist in the same patient, especially co-occur in HIV infected patient. Here, we report a rare case of an AIDS patient who developed eosinophilic folliculitis and Sweet's syndrome within 1 month of initial antiretroviral therapy, presumably due to immune reconstitution inflammatory syndrome. The CD4^+^ T cell counts increased dramatically from 70 to 249 cells/μL within a period of 1 month. Interestingly, the patient was rapidly and strikingly responsive to thalidomide, which has anti-inflammatory, immune regulation, inhibition of neutrophil chemotaxis etc. Moreover, we focused our attention on discussing the clinical, pathological, and possible pathogenic aspects of the rare overlap of HIV complicated with neutrophilic and eosinophilic dermatosis.

## Background

Eosinophilic folliculitis (EF) has been regarded as a significant marker of advanced AIDS (1), reportedly occurs in 9–10% HIV infected patients (2, 3). HIV-associated eosinophilic folliculitis (HIV-EF) was first reported in 1986 by Soeprono and Schinella (4), which is a variant of eosinophilic pustular folliculitis (EPF) (known as Ofuji disease) (5) and characterized by pruritic, erythematous, follicular papules distributed on face, trunk, and limbs. Histopathology of HIV-EF lesion shows eosinophil infiltration in the epithelium of the follicular infundibulum. The specific etiology and pathogenesis of HIV-EF is still unclear. Recently, more reports have suggested HIV-EF is associated with immune reconstitution inflammatory syndrome (IRIS) after commencing anti-retroviral therapy (ART) (6–9), especially when patients with a low baseline CD4 cells count increased rapidly after ART. Maybe due to immune reconstitution recognized antigens from past or ongoing infections (8). Furthermore, accumulated evidence confirmed that Th2 shift and produced cytokines/chemokines play a role in Ofuji disease and a possible pathogenic mechanism of HIV-EF (10), especially interleukin (IL)-4, IL-5, Eotaxin and intercellular adhesion molecule 1 (ICAM-1), which could promote activity, proliferation, and recruitment of eosinophils (11).

Another pro-inflammatory neutrophilic dermatoses (ND) with the predominance of mature neutrophils infiltrate diffusely in the papillary and upper reticular dermis on histopathology, is Sweet's syndrome (also known as acute febrile neutrophilic dermatosis). It can manifest with fever, neutrophilia, tender and painful skin lesions like pseudovesicular nodules and plaques on the face, neck, and upper extremities (12). It was initially proposed by British dermatologist Sweet in 1964 (13). The etiologies and pathogenesis may be multifactorial. It may be associated with tumor antigens (especially hematologic malignancies and underlying malignancy), drugs (granulocyte-colony stimulating factor), infections (bacterial, viral) etc. that can induce cytokine cascade (12). It is classified as classical, drug-induced, and malignancy-associated Sweet's syndrome (14). It has been increasingly reported with co-occurring immunodeficiencies (15, 16). However, Sweet's syndrome has not been well-established associations with HIV. Scattered cases had reported that Sweet's syndrome was associated with IRIS (6, 8, 9). In addition, the immunohistochemistry and serological tests suggested that Th1 (17) and Th17 cells (18) secreted pro-inflammatory cytokines (IL-2, INF-γ, and IL-17) played a dominant role in the pathogenesis of Sweet's syndrome, which could activate and recruit neutrophils to the site of inflammation. Besides, other neutrophil recruiters and activators, such as TNF-α, IL-6, and IL-8 are potential cytokine candidates in the pathogenesis of Sweet's syndrome (14, 19, 20). And some authors have speculated that IL-6 may also be a potential target for the treatment of ND (21).

Both eosinophilic folliculitis and Sweet's syndrome are chronic recurrent and have no specific treatment. But they are sensitive to anti-inflammatory drugs such as glucocorticoids and Non-steroidal anti-inflammatory drugs (NSAIDs) (5, 12). Here, we first reported a case of occurrence of eosinophilic folliculitis and Sweet's syndrome after ART. And first attempted successful treatment with thalidomide and discuss the clinical, pathological, and possible pathogenic aspects of the rare overlap of these three diseases.

## Case Presentation

**Figure 1:**
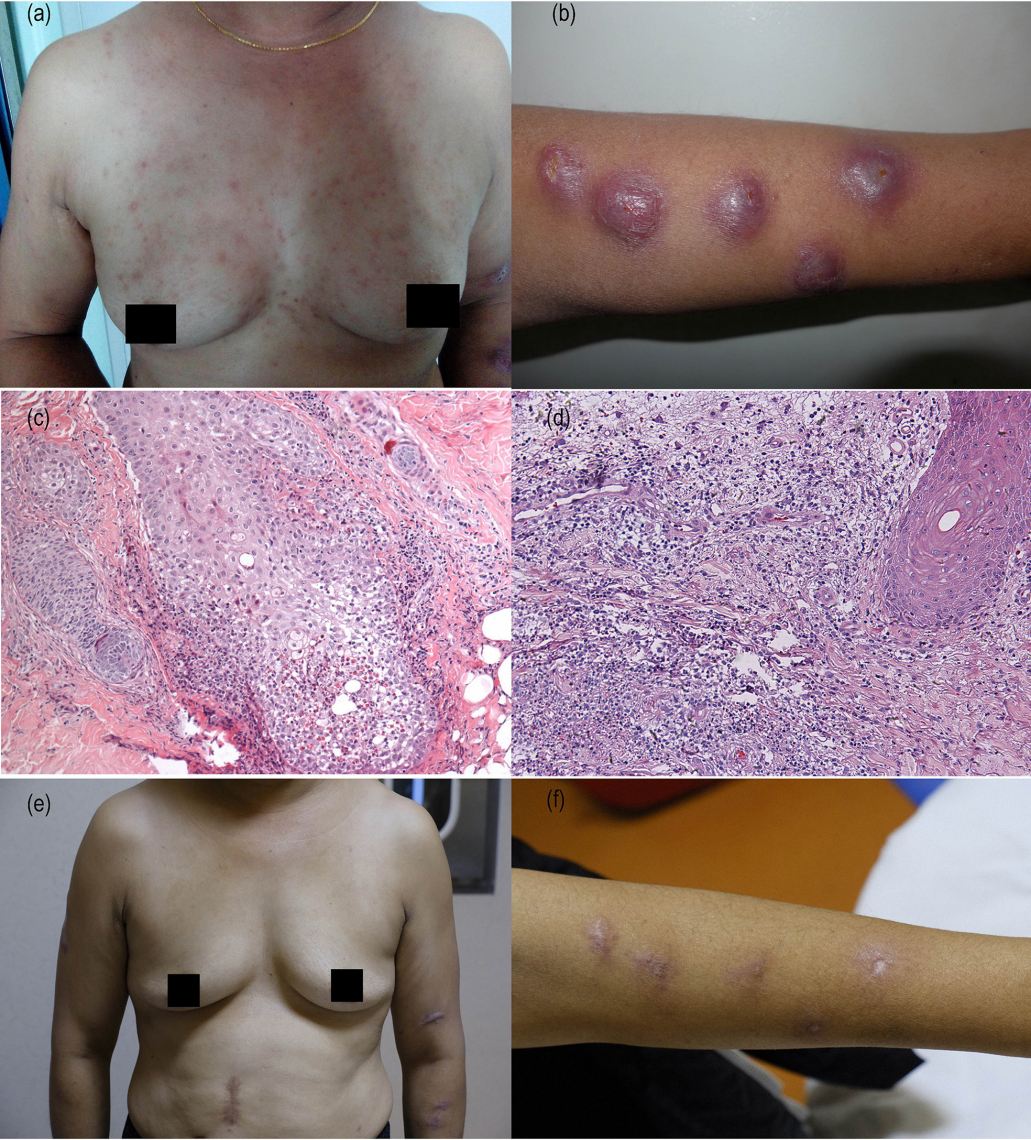
Clinical and histopathological images of the patient before and after treatment. **(a)** Neck and upper chest with erythema and red papules. **(b)** Left forearm with raised, pseudovesicular cysts, and infiltrated plaques. **(c)** Hematoxylin–eosin (HE) staining of skin biopsy from papule at back of neck showing edema of hair follicle epithelial cells in the dermis, surrounded by eosinophils, lymphocytes, and neutrophils inflammatory infiltration. And neutrophils and eosinophils were seen migrated into the hair follicle epithelium (original magnification ×100). **(d)** HE staining of skin biopsy from left forearm plaque showing obvious edema in the superficial dermis, neutrophils, and lymphocytes infiltrated diffusely in the superficial and middle layers of dermis, and nuclear sedimentation were present significantly (×100). **(e)** Neck and upper chest papules resolved completely. **(f)** Forearm with scars formation.

A 47-year-old Chinese woman confirmed HIV-seropositive for 6 years had a history of 3 years of irregular ART, and stopped taking any antiviral medications for the next 3 years because of non-compliance. In September 11 2017, as the CD4^+^ T cells counts was only 70 cells/μL and initiation of ART (tenofovir, lamivudine, and dolutegravir) was started. However, 3 weeks after the onset of ART, there were dense, red follicular papules with itching involving the face, neck and upper trunk, ranging from 2 to 5 mm in diameter (Figure 1a). Routine examination of blood showed white blood cell count of 6.23 × 10^9^/L, the percentage of neutrophils and eosinophils were 58.10% and 8.7%, respectively. The counts of neutrophils and eosinophils were 3.62 × 10^9^/L and 0.54 × 10^9^/L, respectively. She hadn't received any treatment for skin lesions but continued ART.

Then, she had persistent fever for 1 week and the highest temperature was 39°C. She then developed painful, raised, infiltrated plaques, and pseudovesicular on her left forearm (Figure 1b). Re-examination of blood (Table 1) revealed a white blood cell count of 4.54 × 10^9^/L, with 78% neutrophils and 0.4% eosinophils. The neutrophil count was 3.54 × 10^9^/L, the eosinophils count was 0.02 × 10^9^/L; ESR was 68 mm/h, C reactive protein (CRP) was 18.12 mg/L, procalcitonin (PCT) was 0.141 ng/mL and IL-6 was 18.13 pg/ml. The CD4^+^ T lymphocytes counts increased sharply from 70 to 249 cells/μL (Table 1).

**Table 1:** Basic information of the patient.

**Laboratory findings**	**September 11, 2017**	**October 3, 2017**	**October 11, 2017**	**November 12, 2017**
CD4^+^ T (cells/ul)	70	–	249	–
CD8^+^ T (cells/ul)	197	–	577	–
WBC (10^9^/L)	–	6.23	4.54	3.90
Neutrophil (10^9^/L)	—	3.62	3.54	1.97
Neutrophil (%)	–	58.1	78↑	50.6
Eosinophils (10^9^/L)	–	0.54	0.02	0.05
Eosinophils (%)	–	8.7↑	0.4	1.3
IL−6 (pg/ml)	–	–	18.13↑	6.22
CRP(mg/L)	–	–	18.12↑	4.02
ESR(mm/h)	–	–	68↑	12
PCT (ng/ml)	–	–	0.141↑	0.022
(HE) staining	–	–	Confirmed Sweet's syndrome and HIV–EF	–

*WBC, white blood cell; IL, interleukin; CRP, C–Reactive Protein; ESR, erythrocyte sedimentation rate; PCT, procalcitonin*.

She underwent dermatopathology biopsies in two sites from her left forearm plaque and the back of neck papules. The pathological examination of the papules confirmed the diagnosis of eosinophilic folliculitis, edema of hair follicle epithelial cells in the dermis, surrounded by eosinophils, lymphocytes, and neutrophils inflammatory infiltration. And neutrophils and eosinophils were seen migrated into the hair follicle epithelium (Figure 1c). Pathological examination of the left forearm biopsy confirmed the diagnosis of Sweet's syndrome with obvious edema in the superficial dermis, neutrophils and lymphocytes infiltrated diffusely in the superficial and middle layers of dermis, and nuclear sedimentation were present significantly (Figure 1d). No obvious abnormality was found in the examination of lung, liver, renal etc. and without any other systemic manifestations.

Considering the CD4^+^ T cells count increased sharply, the IL-6 and C-reactive protein in peripheral blood were increased. Combined with clinical manifestations and pathological examination results, the patient was diagnosed as AIDS complicated with Sweet's syndrome and eosinophilic folliculitis. We speculate that the patient's symptoms were attributable to IRIS. The patient received thalidomide 100 mg/day combined with ART. On follow-up half month later, the follicular papules on face, neck and trunk were improved and about 1 month after the onset of treatment the plaques were also improved significantly (Figures 1e,f). The patient developed scars at the site of plaque on the forearm after treatment and are present till now. Re-examination laboratory tests showed CRP was 4.02 mg/L, PCT was 0.022 ng/mL and IL-6 was 6.22 pg/ml, all of which returned to their normal levels. The blood tests returned to normal (Table 1). ART was continued during the treatment of thalidomide. There is no sign of relapsing pruritic papules and plaque up till now.

## Discussion

Anecdotally, Sweet's syndrome and eosinophilic folliculitis co-occurring with immunodeficiencies is very rare. It may be related through an overlap in immunopathogenesis. There are different postulated pathogeneses for them, but the patient suffered Sweet's syndrome followed by EF successively within 1 month of initial ART, accompanied by a sharp increase in CD4^+^ T cells. It indicated that her immunity state switched from disease of predominant Th2 activation—EF—to disease of predominant Th1 activation—Sweet's syndrome. There seems to be a shift of Th2 cytokines production and a decline in Th1 cytokines as CD4^+^ T cells decreased with AIDS progression (22), which may first contribute to EF, and then developed Sweet's syndrome with increase in Th1 cytokines secretion. In addition, rapid recovery of effector memory CD4^+^T cells in the first phase of CD4 cell recovery (23) also restored immunity to previously non-pathogenic infectious state (8, 14), possibly resulting in Sweet's syndrome and eosinophilic folliculitis. It still needs to be explored whether the rapidly increased and newly distributed CD4^+^ T cells could directly or indirectly regulate neutrophils and eosinophils and inflammatory agents and may contribute to Sweet's syndrome and EF.

ART restores the Th1/Th2 cell balance, which allows the adaptive immune system to overcome some dermatosis. However, new appearance or paradoxical worsening of existing dermatosis may attribute to dramatic immune restoration because of IRIS which occurs in about 15–25% of patients in the early stage of ART (24). IRIS is the excessive inflammatory response to the existing pathogens or underlying antigens in the early stage of ART. The increased levels of IL-6, TNF-a, and CRP can be potential biomarkers for IRIS (25, 26). The rapidly increased of circulating CD4 cells leads to the increase of Th1 and Th2 cells and their secreted cytokines (IL-4, IL-5, IL-6, IL-8, TNF-a ect) (22), which may promote the occurrence and development of Sweet's syndrome and eosinophilic folliculitis. Taken together, we speculate that the presence of Sweet's syndrome and EF after ART may be closely related to IRIS. Although it has been reported that ART drug abacavir can cause Sweet's syndrome (27). We affirmed that the patient suffered Sweet's syndrome was not caused by ART drugs, because the patient's skin lesions improved when the ART was not stopped during the treatment of thalidomide. At the same time, comprehensive laboratory tests have also ruled out malignancy related Sweet's syndrome.

There are many methods of treatment for HIV-EF and Sweet's syndrome, co-treatment included anti-inflammatory agents of glucocorticoids and NSAIDs. Thalidomide is a strong non-steroid anti-inflammatory drug and immunomodulatory which seems to have been forgotten or abandoned due to the side effects of “seal-leg deformity” since 1961 (28). Thalidomide is contraindicated in pregnant females because of its adverse teratogenic side effects. Prolonged administration of thalidomide can cause peripheral neuropathy, drowsiness and fatigue (29). In addition, venous thrombosis, neutropenia and cardiovascular side effects have also been reported in clinical practice (30). Therefore, baseline electrophysiological examinations, coagulation function, absolute neutrophil count, and cardiac function should be monitored carefully during thalidomide treatment.

Recently, it has been approved and used in the field of dermatology, such as leprosy nodular erythema, vascular lupus erythematosus, and refractory inflammatory bowel disease (29). Although thalidomide had been reported for treatment of HIV/AIDS-related oral ulcers (30) or Kaposi's sarcoma (31). But there have been no reports concerning thalidomide treatment for AIDS associated Sweet's syndrome or EF. Only one report on thalidomide had achieved good curative effect without relapse after ineffective systemic corticosteroids therapy in Sweet's syndrome associated with myelodysplasia (32).

Thalidomide could inhibit TNF-a, IL-1β, and IL-6 production (33, 34). This benefits not only by inhibiting SS and EF associated cytokines (IL-4, IL-5, IL-6, IL-8, TNF-a etc.), but also by inhibiting IRIS induced release of inflammatory cytokines (TNF-a, IL-6 etc.). More importantly, pro-inflammatory cytokine TNF-a could not only mediate the recruitment and activation of eosinophils by expressing adhesion molecules such as ICAM-1, but also activates and chemotactic neutrophils directly (35). Therefore, the use of TNF-a inhibitor infliximab or etanercept has been effective in the treatment of EF and Sweet's syndrome (36, 37). In addition, TNF-α induces viral replication via NF-κB-dependent transcriptional pathway (38), the anti-TNF activity of thalidomide may reduce HIV viral replication (39), which also benefits HIV infected patients. In this case, it significantly improved HIV associated with eosinophilic and neutrophilic dermatoses lesions, which means another weapon to be considered in the novel therapeutic strategies.

To our knowledge, this was the first report of AIDS patient co-incidental occurrence of Sweet's syndrome and eosinophilic folliculitis. Moreover, it was the first to report on the efficacy of old drug thalidomide for treatment of HIV-EF and Sweet's syndrome. This case provides a new perspective for the dialectical understanding of pharmaceutical value of thalidomide rather than its complete negation. Thalidomide may be an effective and safe treatment strategy for HIV patients with eosinophilic and (or) neutrophilic dermatitis.

## Ethics Statement

The studies involving human participants were reviewed and approved by the studies involving human participants were reviewed and approved by The Ethics committe of First Affiliated Hospital of Kunming Medical University. The patients/participants provided their written informed consent to participate in this study. The patients/participants provided their written informed consent to participate in this study.

## Author Contributions

R-JD, Y-YL, and Y-JZ contributed conception and design of the study. S-ZH performed the statistical analysis. SS wrote the first draft of the manuscript. PU wrote sections of the manuscript. All authors contributed to manuscript revision, read and approved the submitted version.

### Conflict of Interest

The authors declare that the research was conducted in the absence of any commercial or financial relationships that could be construed as a potential conflict of interest.
